# A tale of two waves: characteristics and outcomes of COVID-19 admissions during the Omicron-driven fourth wave in Cape Town, South Africa, and implications for the future

**DOI:** 10.1016/j.ijregi.2022.11.008

**Published:** 2022-11-24

**Authors:** Muhammad Saadiq Moolla, Tongai Gibson Maponga, Haroon Moolla, Eve Kollenberg, Samantha Anie, Aisha Moolla, Desiree Moodley, Usha Lalla, Brian W Allwood, Neshaad Schrueder, Wolfgang Preiser, Coenraad FN Koegelenberg, Arifa Parker

**Affiliations:** a Division of Pulmonology, Department of Medicine, Faculty of Medicine and Health Sciences, Stellenbosch University and Tygerberg Hospital, Cape Town, South Africa; b Division of Medical Virology, University of Stellenbosch and National Health Laboratory Service, Cape Town, South Africa; c Centre for Infectious Disease Epidemiology and Research, School of Public Health, Faculty of Health Sciences, University of Cape Town, Cape Town, South Africa; d Division of General Medicine, Department of Medicine, Faculty of Medicine and Health Sciences, Stellenbosch University and Tygerberg Hospital, Cape Town, South Africa; e SAMRC/Wits Centre for Health Economics and Decision Science — PRICELESS SA, School of Public Health, Faculty of Health Sciences, University of the Witwatersrand, Johannesburg, South Africa; f National Health Laboratory Service, Tygerberg Business Unit, Cape Town, South Africa; g Division of Infectious Diseases, Department of Medicine, Faculty of Medicine and Health Sciences, Stellenbosch University and Tygerberg Hospital, Cape Town, South Africa

**Keywords:** COVID-19, SARS-CoV-2, Omicron variant, fourth wave, lockdown, pandemic restrictions

## Abstract

**Objectives:**

The aim of this study was to describe the pattern of admissions during the fourth wave of COVID-19 in order to inform future public health policies.

**Methods:**

This was a retrospective descriptive study of an early cohort of all adult patients with SARS-CoV-2 infection admitted to a tertiary hospital in Cape Town, South Africa, at the start of the country's fourth wave. This was compared with an early cohort from the first wave at the same institution.

**Results:**

In total, 121 SARS-CoV-2-positive admissions from the fourth wave were included. Thirty-one (25.6%) patients had COVID-19 pneumonia, while 90 (74.4%) had incidental SARS-CoV-2 infection. (In the first wave all 116 patients had COVID-19 pneumonia.) Thirty-two (26.4%) patients self-reported complete or partial COVID-19 vaccination, of whom 12 (37.5%) were admitted with COVID-19 pneumonia. Compared with the first wave, there were fewer intensive- or high-care admissions (18/121 [14.9%] vs 42/116 [36.2%]; *p* < 0.001) and mortality was lower (12/121 [9.9%] vs 31/116 [26.7%]; *p* = 0.001).

**Conclusion:**

Admissions to the COVID-19 wards during the fourth wave primarily included patients with incidental SARS-CoV-2 infection. There was a reduction in the need for critical care and in-hospital mortality. This changing epidemiology of COVID-19 admissions may be attributed to a combination of natural and/or vaccination-acquired immunity.

## Highlights

• A reduction in COVID-19 pneumonia admissions was observed during the fourth wave.
• Requirements for ICU and mortality rates were lower than during the first wave.
• The findings support de-escalating COVID-19 public health measures in certain settings.

## Introduction

South Africa was one of the first countries to implement public health measures early in the trajectory of the coronavirus disease 2019 (COVID-19) pandemic. These included a hard lockdown during the first wave as well as early implementation of a universal mask-wearing strategy (Broadbent et al., 2020; Moonasar et al., 2021). These measures were impactful during the first wave of COVID-19 driven by the D614G variant (Reichert et al., 2022). The country subsequently experienced heavy second and third waves driven by the beta and delta variants, respectively (Cohen et al., 2022) with over 100 000 deaths nationally in each wave (Bradshaw et al., 2022).

The Omicron variant, first detected in South Africa, was responsible for driving the fourth wave in the country between November 2021 and January 2022 (Viana et al., 2022). At this time, 46% of the general public at risk had been vaccinated (Mendelsohn et al., 2022). Household transmission surveys (Cohen et al., 2022) and blood transfusion surveys (Cable et al., 2022) also showed that a significant proportion of the population had experienced prior natural infection, likely leading to immunity.

Our study aimed to describe the clinical characteristics and outcomes of an early cohort of COVID-19 patients admitted during the fourth wave. These were compared with those of an early cohort of patients admitted in the first wave, who were infected with the original strain, were not vaccinated, and had no prior infection.

## Methods

### Study design and population

This single-centre retrospective descriptive study included an early cohort of all consecutive patients aged ≥ 13 years with a SARS-CoV-2-positive polymerase chain reaction or rapid antigen result admitted to the designated COVID-19 wards at Tygerberg Hospital (TBH), Cape Town, South Africa, during December 1–25, 2021, coinciding with the start of the fourth wave of COVID-19 admissions at our institution. Results were compared with those for a previously reported early cohort of patients admitted during the first wave (Parker et al., 2020). The prior study used the same recruitment and data collection strategies.

### Study setting

TBH is a 1380-bed academic hospital in the Western Cape province of South Africa that provides a tertiary service to a population of approximately 3.5 million. At the start of the pandemic, any patient testing positive for SARS-CoV-2 and requiring inpatient management was accepted from primary- and secondary-level hospitals in the drainage area. By the fourth wave, only severely ill patients requiring at least non-rebreather oxygen were routinely transferred.

### General patient management

Most patients were tested for SARS-CoV-2 infection via a nasopharyngeal swab. When available, other respiratory specimens, such as sputum or tracheal aspirates, were also tested. During the fourth wave, patients with severe COVID-19 were managed with intravenous dexamethasone (RECOVERY Collaborative Group, 2021) while milder cases requiring nasal prong or facemask oxygen were treated with oral prednisone. Therapeutic anticoagulation with enoxaparin (Barnes et al., 2020) was also administered, except in milder cases. Antibiotics were not routinely or empirically prescribed (Moolla et al., 2021). Oxygenation support was escalated from nasal prong and facemask oxygen to high-flow nasal cannula, followed by intubation and mechanical ventilation as per clinical indications (Lalla et al., 2020).

This differed from the first wave, which occurred prior to the RECOVERY trial (RECOVERY Collaborative Group, 2021), where no patients received steroids. During this time, patients were managed with oxygen support, prophylactic doses of enoxaparin, and empiric antibiotics until the diagnosis of COVID-19 was confirmed.

### Inpatient screening

Patients who were asymptomatic for SARS-CoV-2 but admitted to TBH for other indications underwent screening at the discretion of the treating team. Different patterns of screening occurred in different departments. Indications for screening included patients being unable to give a history, preoperatively or prior to transfer to other facilities. Incidental SARS-CoV-2 referred to asymptomatic patients who tested positive but did not have clinical or radiological evidence of COVID-19 pneumonia, and who required admission for indications unrelated to COVID-19. Such patients were admitted to the COVID-19 wards for isolation.

### Data collection

Patients admitted to COVID-19 wards were identified through a review of nursing and administrative records in each ward. Data were extracted from hospital records and laboratory results, using a standardized form, and included demographic details, symptoms, comorbidities, laboratory results, management, length of admission, and outcome (death or discharge). CD4 cell count, human immunodeficient virus (HIV) viral load, and glycated hemoglobin (HbA1c) were captured on admission or within 6 months prior to admission. Obesity and body mass index (BMI) were documented on the standardized admission tool at the discretion of the primary caregivers, either based on the calculated BMI or the clinician's impression.

Patients were followed up until discharge, transfer from hospital, or death.

### Sequencing

Whole-genome sequencing of SARS-CoV-2 was performed using the Midnight Protocol (Oxford Nanopore Technologies, Oxford, UK) (Freed and Silander, 2021), from archived eluates obtained from nasopharyngeal swabs, as previously described. The assignment of clades and identification of mutations were performed using Nextclade (Aksamentov et al., 2021) and lineages were determined using the web version of the Phylogenetic Assignment of Named Global Outbreak Lineages (PANGOLIN) software (O'Toole et al., 2021).

### Statistical analyses

The cohort was compared with a control cohort of patients admitted to our institution at the start of the first wave of SARS-CoV-2 infections in South Africa (Parker et al., 2020). Differences between the cohorts were assessed by univariate analyses. The chi-square test, Fisher's exact test, and Mann–Whitney U-test were used, as appropriate, and *p*-values less than 0.05 were regarded as statistically significant. Descriptive numerical data were summarized using medians and interquartile ranges (IQRs). All analyses were performed using Stata version 13.1 (StataCorp, Texas, USA).

### Ethical approval

This study was approved by the Health Research Ethics Committee of Stellenbosch University (ref. N20/04/002_COVID-19).

## Results

### Baseline data

**Figure 1:**
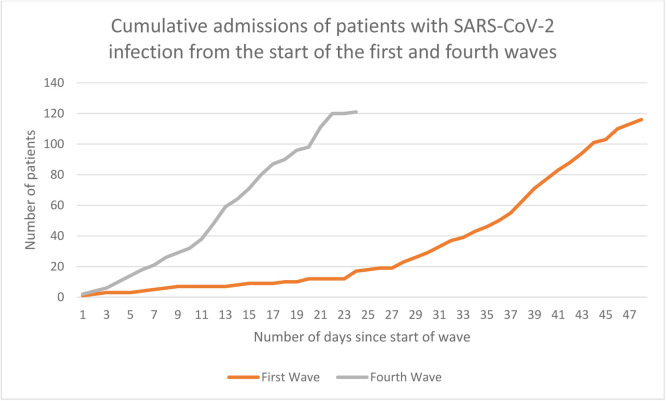
Cumulative admission of patients with SARS-CoV-2 infection from the start of the first and fourth waves at Tygerberg Hospital, Cape Town, South Africa.

In total, 121 patients with SARS-CoV-2 infection admitted to the COVID-19 wards during the study period were identified (Figure 1). Of these, 31 had COVID-19 pneumonia and the rest were asymptomatic or incidental cases. All patients were included in the analysis. The first-wave control cohort included 116 patients, all of whom had COVID-19 pneumonia (Parker et al., 2020).

The median age of patients admitted was 35 (IQR 26–55) years, significantly lower than that of the first wave cohort (median 49, IQR 39–59; *p* < 0.001). Patients in the fourth wave were less likely to have hypertension (17.4% vs 39.7%; *p* = 0.03) or diabetes mellitus (17.4% vs 37.1%; *p* < 0.001) compared with patients in the first wave. However, no statistically significant difference was found when considering the subset of patients with COVID-19 pneumonia.

The majority of patients were female (fourth wave 64.5% vs first wave 61.2%). The study found higher rates of obesity (43.8% vs 27.6%; *p* = 0.014), vascular disease (4.1% vs 0.0%; *p* = 0.029), smoking (28.1% vs 9.5%; *p* < 0.001), and pregnancy (20.7% vs 4.3%, *p* < 0.001) among patients admitted during the fourth wave compared with the first (Table 1 and Supplementary Table 1).

**Table 1:** Baseline laboratory and outcome data for patients admitted during the fourth and first waves.

Characteristic	Fourth-wave cohort (pneumonia)	Fourth-wave cohort (incidental)	Fourth-wave cohort (all)	First-wave cohort	*p*-value*
Number of patients	31	90	121	116	
Baseline					
Age (years)	53 (33–67)	32 (25–46)	35 (26–55)	49 (39–59)	< 0.001
Sex (male)	14 (45.2)	29 (32.2)	43 (35.5)	45 (38.8)	0.604
Hypertension	11 (35.5)	22 (24.4)	33 (27.3)	46 (39.7)	0.03
Diabetes mellitus	12 (38.7)	9 (10.0)	21 (17.4)	43 (37.1)	< 0.001
Obesity	13 (41.9)	40 (44.4)	53 (43.8)	32 (27.6)	0.014
BMI (kg/m^2^)	24.2 (22.0–29.4)	24.7 (22.2–29.4)	24.7 (22.2–29.4)	#	
HIV	4 (12.9)	16 (17.8)	20 (16.5)	24 (20.7)	0.74
CD4 cell count	$	179 (88–235)	130 (76–231)	279 (160–492)	0.013
HIV viral load (copies/mL)	$	171 (33–634104)	4439 (42–797 098)	< 1000	
TB					
current	2 (6.5)	5 (5.6)	7 (5.8)	4 (3.4)	0.426
Previous	0 (0)	5 (5.6)	5 (4.1)	9 (7.8)	0.21
Smoking history	12 (38.7)	22 (24.4)	34 (28.1)	11 (9.5)	< 0.001
Pregnant	1 (3.2)	24 (26.7)	25 (20.7)	5 (4.3)	< 0.001
Previous COVID-19					
confirmed	1 (3.2)	3 (3.3)	4 (3.3)	N/A	
suspected	1 (3.2)	2 (2.2)	3 (2.5)	N/A	
Vaccination					
complete	8 (25.8)	14 (15.6)	22 (18.2)	N/A	
partial	4 (12.9)	6 (6.7)	10 (8.3)	N/A	
either	12 (38.7)	20 (22.2)	32 (26.4)	N/A	
Laboratory values					
Oxygen saturation (%)	94 (88–98)	97 (96–99)	97 (93–99)	94 (90–97)	< 0.001
PaO_2_ (kPa)	9.3 (6.5–14.8)	12.8 (10.2–16.8)	11.7 (8.9–16.2)	8.7 (7.2–11.9)	< 0.001
FiO_2_ (%)	40 (21–80)	21 (21–21)	21 (21–33)	21 (21–40)	< 0.001
P:F ratio	170 (111–332)	415 (287–539)	360 (180–511)	246 (137–357)	0.004
Creatinine (mmol/L)	77 (56–91)	68 (53–93)	72 (54–92)	75.5 (57.5–113.5)	0.241
WCC (× 10^9^/L)	8.3 (6.1–11.7)	8.9 (6.7–12.9)	8.8 (6.3–12.7)	7.6 (6.2–9.9)	0.017
Neutrophil count (× 10^9^/L)	7.9 (4.6–9.9)	6.7 (4.8–10.3)	6.8 (4.7–10.0)	6.0 (4.2–7.8)	0.024
Lymphocyte count (× 10^9^/L)	0.9 (0.6–1.4)	1.1 (0.7–1.5)	1.1 (0.7–1.4)	1.1 (0.8–1.7)	0.202
CRP (mg/L)	134 (45–221)	66 (15–169)	78 (20–190)	138 (63–222)	0.008
LDH	409 (313–627)	454 (316–564)	453 (307–582)	446 (288–606)	0.797
D-dimer (mg/L)	1.5 (0.5–5.4)	1.9 (1.0–3.0)	1.8 (0.6–5.0)	0.6 (0.3–1.1)	0.029
Outcomes					
Highest level of care					
General ward	26 (83.9)	77 (85.6)	103 (85.1)	74 (63.8)	< 0.001
ICU/HCU	5 (16.1)	13 (14.4)	18 (14.9)	42 (36.2)	< 0.001
Hospital stay (days)	5 (3–7)	7 (3–12)	6 (3–10)	6 (3–10)	0.548
Outcome					
Survived	27 (87.1)	76 (84.4)	103 (85.1)	85 (73.3)	0.024
Deceased	3 (9.7)	9 (10.0)	12 (9.9)	31 (26.7)	0.001

Table values are number (percent) or median (interquartile range).

⁎ Comparing fourth-wave cohort (all) and first-wave cohort.

# No data

$ ≤ three values CRP = C-reactive protein, F_i_O_2_ = fraction of inspired oxygen, HCU = high-care unit, HIV = human immunodeficiency virus, ICU = intensive-care unit, LDH = lactate dehydrogenase, P:F ratio = ratio of FiO_2_ to PaO_2_, PaO_2_ = partial pressure of oxygen, TB = tuberculosis, WCC = white cell count

Few patients had a history of laboratory-confirmed (*n* = 4) or self-reported (*n* = 3) SARS-CoV-2 infection prior to their admission during the fourth wave, and 32/121 (26.5%) patients reported partial or complete vaccination.

### Risk factors for COVID-19 pneumonia

Amongst the fourth-wave cohort, patients with COVID-19 pneumonia were older (median age 53 years, IQR 33–67) compared with those with incidental SARS-CoV-2 infection (median age 32 years, IQR 25–46; *p* < 0.001). They also had a higher prevalence of diabetes mellitus (38.7% vs 10.0%; *p* < 0.001), cardiac disease (19.4% vs 9.1%; *p* = 0.021), and vascular disease (16.1% vs 4.1%; *p* < 0.001), as shown in Table 2.

**Table 2:** Comparison of comorbidities between patients with COVID-19 pneumonia and incidental SARS-CoV-2 during the fourth wave at Tygerberg Hospital, Cape Town, South Africa

Risk factor	COVID-19 pneumonia	Incidental SARS-CoV-2	*p*-value*
Number of patients	31	90	
Age (years)	53 (33–67)	32 (25–46)	< 0.001
Sex (male)	14 (45.2)	29 (32.2)	0.194
Hypertension	11 (35.5)	22 (24.4)	0.234
Diabetes mellitus	12 (38.7)	9 (10.0)	< 0.001
Cholesterol	6 (19.4)	7 (7.8)	0.073
Obesity	13 (41.9)	40 (44.4)	0.808
Cardiac disease	6 (19.4)	5 (5.6)	0.021
Vascular disease	5 (16.1)	0 (0.0)	< 0.001
Malignancy	3 (9.7)	2 (2.2)	0.072
HIV	4 (12.9)	16 (17.8)	0.718
TB			
current	2 (6.5)	5 (5.6)	0.854
previous	0 (0)	5 (5.6)	0.180
either	2 (6.5)	10 (11.1)	0.405
Other lung disease	6 (19.4)	8 (8.9)	0.343
CTD	0 (0)	1 (1.1)	0.556
CKD	1 (3.2)	6 (6.7)	0.682
Smoking history	12 (38.7)	22 (24.4)	0.128
Alcohol	6 (19.4)	19 (21.1)	0.897
Pregnant	1 (3.2)	24 (26.7)	0.005

Table values are number (percent) or median (interquartile range).

CTD = connective tissue disease, CKD = chronic kidney disease, HIV = human immunodeficiency virus, TB = tuberculosis

There was a lower prevalence of pregnancy in the group with COVID-19 pneumonia (3.2%) compared with those with incidental SARS-CoV-2 infection (26.7%; *p* = 0.005). There was no significant difference in the prevalence of male sex (45.2% vs 32.2%; *p* = 0.194), hypertension (35.5% vs 24.4%; *p* = 0.234) or obesity (41.9% vs 44.4%, *p* = 0.808) between the two groups. There was also no significant difference in the prevalence of HIV (12.9% vs 17.8%; *p* = 0.718) or pulmonary tuberculosis (6.5% vs 5.6%, *p* = 0.854) between the two groups.

### Clinical and laboratory features

Fewer patients reported symptoms of coughing, shortness of breath, fever (all *p* < 0.001), loss of smell (*p* = 0.045), chest pain (*p* = 0.007), and myalgia (*p* = 0.009) during the fourth wave, but these differences were not seen when considering only the subset of patients with COVID-19 pneumonia. Malaise was more common during the fourth wave (24.8% vs 13.8%, *p* = 0.041). Further details are shown in Supplementary Table 1.

Patients admitted during the fourth wave had significantly higher oxygen saturation and arterial partial pressure of oxygen (*p* < 0.001) compared with those in the first wave. This difference was not seen in the COVID-19 pneumonia subset. Similarities and differences in other results are shown in Table 1 and Supplementary Table 1.

A higher proportion of patients received only ward-level care during the fourth wave, with 14.9% of patients admitted to intensive or high care compared with 36.2% in the first wave (*p* < 0.001).

### Sequencing

In the fourth wave cohort, 57 samples were available for sequencing. Sequencing failed on 15 specimens because of high Ct values, which are indicative of a low viral load in the specimen. Of the 42 sequenced specimens, 41 (97.6%) were found to be the Omicron variant (40 with BA.1 lineage and one with BA.2) and one was found to be the Delta variant (AY.32).

### Outcomes

Mortality at our institution was significantly lower in the fourth wave (*n* = 12/121, 9.9%) compared with the first wave (*n* = 31/116, 26.7%; p=0.001). Similarly, mortality was lower in the COVID-19 pneumonia subset of the fourth wave (*n* = 3/31, 9.7%; p = 0.05) compared with the first wave.

### Effects of vaccination

Twelve (38.7%) patients with COVID-19 pneumonia during the fourth wave self-reported either partial or complete vaccination, while 20 (22.2%) patients with incidental SARS-CoV-2 reported the same. Patients with partial or complete vaccination had a higher baseline creatinine level (82 mmol/L [IQR 67–95] vs 63 mmol/L [IQR 51–83]; *p* = 0.012) compared with those who reported being unvaccinated. Aside from this finding, there was no significant association between reported vaccination status and the occurrence of incidental SARS-CoV-2 infection or COVID-19 pneumonia, various recorded indices of disease severity, or patient outcomes during the Omicron-driven fourth wave (Table 3).

**Table 3:** Association between reported vaccination status and diagnosis of incidental SARS–CoV–2 infection (as opposed to COVID–19 pneumonia), severity indices, and patient outcomes during the Omicron–driven fourth wave

Characteristic	Partial or complete vaccination	No vaccination	*p*–value
Diagnosis			
Incidental COVID-19	20 (62.5)	61 (77.2)	0.114
Severity indices			
Oxygen saturation (%)	96 (92–99)	98 (96–99)	0.157
PaO_2_ (kPa)	10.8 (8.6–15.1)	13.0 (10.1–16.9)	0.255
FiO_2_ (%)	21 (21–21)	21 (21–33)	0.657
P:F ratio	329 (190–404)	411 (172–539)	0.346
Creatinine (mmol/L)	82 (67–95)	63 (51–83)	0.012
WCC (× 10^9^/L)	7.9 (6.1–11.3)	9.3 (6.5–13.0)	0.318
Neutrophil count (× 10^9^/L)	5.9 (4.6–8.2)	7.7 (4.9–12.6)	0.138
Lymphocyte count (× 10^9^/L)	0.87 (0.65–1.19)	1.26 (0.74–1.80)	0.058
CRP (mg/L)	97 (12–229)	72 (19–176)	0.626
LDH	417 (331–518)	429 (284–584)	0.751
D-dimer (mg/L)	$	1.6 (0.5–3.3)	N/A
Outcomes			
Hospital stay (days)	6 (4–8)	5 (3–10)	0.986
Need for ICU/HCU	3 (9.4)	11 (13.9)	0.513
Deceased	4 (12.5)	6 (7.6)	0.414

Table values are number (percent) or median (interquartile range).

$ ≤ three values CRP = C-reactive protein, FiO_2_ = fraction of inspired oxygen, HCU = high-care unit, ICU = intensive-care unit, LDH = lactate dehydrogenase, P:F ratio = ratio of FiO_2_ to PaO_2_, PaO_2_ = partial pressure of oxygen, WCC = white cell count

## Discussion

The early fourth wave of SARS-CoV-2 infections in our region was driven by the Omicron variant (BA.1 lineage). There was a much faster rise in admissions to the COVID-19 wards during this time compared with the first wave, led principally by the incidental detection of SARS-CoV-2 infection in asymptomatic and mild cases that were admitted to hospital for unrelated indications. Patients were less likely to have a primary diagnosis of COVID-19 pneumonia, and there was a reduction in hospital admission for COVID-19 pneumonia compared with background infection rates (Mendelsohn et al., 2022). There were fewer cases of pneumonia, a reduction in the need for critical care, and reduced mortality in the fourth wave compared with the first wave, including within the subgroup of patients with COVID-19 pneumonia.

The faster rise in admissions to the COVID-19 wards at the start of the fourth wave was likely due to the country remaining at a limited ‘Level 1’ lockdown during this time, compared with the full ‘Level 5’ lockdown at the start of the first wave. This resulted in wider community spread during the fourth wave at a time when non-COVID-19 services at the hospital remained open, resulting in increased admission of asymptomatic and mild cases to the COVID-19 wards (Broadbent et al., 2020; Moonasar et al., 2021).

The finding of reduced severity may be explained by a combination of differences in the virulence of the virus variants, vaccination coverage, and natural immunity due to previous infection. Few patients (5.5%) reported a personal history of previous SARS-CoV-2 infection, despite high reported rates of previous infection in local studies (Cable et al., 2022; Cohen et al., 2022). This suggests a degree of protection due to previous infection. However, given that asymptomatic infection (and thus recall bias) was possible, care needs to be taken in drawing this conclusion.

Reported vaccination rates in this study were low, and prior vaccination did not appear to confer any benefit in terms of admission with incidental SARS-CoV-2 as opposed to COVID-19 pneumonia, or lower indices of severity of disease or outcome. Reasons for this may be the waning of immunity due to the time elapsed since vaccination, the large proportion with partial vaccination (10/32), passive immunity due to prior infection, lack of statistical power to detect differences, and confounding due to indications for hospital admission, where other disease processes caused severe illness and poor outcomes.

Evidence of some degree of protection conferred by vaccination was that only 26.4% of the cohort reported complete or partial vaccination, while 46% of adults in the Western Cape were completely vaccinated in December 2021 (Mendelsohn et al., 2022). At the time of writing, South Africa remained short of its target to vaccinate at least 67% of its population. After initial hurdles with procurement and roll-out of COVID-19 vaccines were overcome, vaccine hesitancy related to misinformation and concerns about safety and effectiveness have emerged as a significant challenge in reaching this goal. Poor messaging regarding vaccines has exacerbated the problem. Governmental and civil society campaigns have sought to address these problems (Cooper et al., 2021).

Previously reported risk factors for severe COVID-19, namely older age, diabetes mellitus, and cardiovascular disease, remained significantly more prevalent in the pneumonia group compared with the incidental group. Male sex and hypertension also had higher prevalences, although not reaching statistical significance. These risk factors remain relevant for targeted vaccination campaigns as we move out of the pandemic phase.

Obesity has emerged as a significant risk factor for COVID-19 morbidity and mortality (Parker et al., 2022). Although a higher proportion of patients with obesity was found during the fourth wave, this finding should be interpreted with caution since it may be due to better awareness and reporting rather than a real increase. Notably there was no significant difference in obesity rates between the COVID-19 pneumonia group and the incidental SARS-CoV-2 group during the fourth wave.

While this study describes findings at a single hospital, reports from other hospitals in South Africa (Jassat et al., 2021; Abdullah et al., 2022; Mendelsohn et al., 2022) have revealed similar findings during the fourth wave, where COVID-19 wards were mostly filled with mild and incidental cases requiring minimal oxygenation, as opposed to previous waves where patients were sicker and oxygen requirements were much higher.

The limitations of this study include its retrospective nature and small subgroup sizes. A significant challenge in this study comparing cohorts from two different waves of COVID-19 was the variation in the management received by patients. During the fourth wave, patient management was informed by experience gained during prior waves, as well as evidence from randomized control trials. On the other hand, far fewer resources were allocated to the COVID-19 response compared with the first wave, with fewer ward beds and staff available, and differing referral patterns. This makes it difficult to determine the extent to which the reduction in mortality was related to improved patient care, passive or vaccine-induced immunity, or the potential reduction in virulence of newer SARS-CoV-2 variants. It is likely a combination of some of these factors.

## Conclusion

During the fourth wave, driven by the Omicron BA.1 variant, admissions to the COVID-19 wards primarily included patients with incidental SARS-CoV-2 infections. Conversely, all patients were admitted with COVID-19 pneumonia during the first wave. In the fourth wave there was a reduction in the number of pneumonia cases, the need for critical care, and in-hospital mortality. Vaccination uptake and prior history of self-reported infection in admitted patients were found to be low, suggesting a degree of protection. The changing epidemiology of COVID-19 admissions supports the relaxation of COVID-19 public health measures in similar settings with high vaccination coverage or natural infection rates. Previously reported risk factors for disease severity remain relevant and can inform targeted vaccination campaigns going forward.

## Declarations

None

## Author contributions

MSM and AP conceptualized the study and drafted the manuscript. MSM, EK, and SA collected the data. TM and WP performed the sequencing. HM and AM analyzed the data. All authors contributed to and approved the final manuscript.

## Funding sources

None

## Ethical approval

The study was approved by the Health Research Ethics Committee of Stellenbosch University (ref. N20/04/002_COVID-19).

## Conflicts of interest

None

## Appendix Supplementary materials

Image, application 1
