# Supplementary material for: A tale of two waves: characteristics and outcomes of COVID-19 admissions during the Omicron-driven fourth wave in Cape Town, South Africa, and implications for the future
Source: IJID Reg. 2022 Nov 24;6:42–7. doi: 10.1016/j.ijregi.2022.11.008 (PMC9684091; doi:10.1016/j.ijregi.2022.11.008)
Supplement: Supplementary file 1 [file mmc1.docx]

Supplementary Table 1. Baseline, clinical, laboratory, and outcome data for COVID–19 pneumonia patients admitted during the fourth wave, patients with incidental SARS–CoV–2 infection admitted during the fourth wave, all patients admitted during the fourth wave, and the early cohort of patients admitted during the first wave

| **Characteristic** | **Fourth-wave cohort (pneumonia)** | **Fourth-wave cohort (incidental)** | **Fourth-wave cohort (all)** | **First-wave cohort** | ***p*-value*** |
| --- | --- | --- | --- | --- | --- |
| Number of patients | 31 | 90 | 121 | 116 |  |
| **Baseline** |  |  |  |  |  |
| Age (years) | 53 (33–67) | 32 (25–46) | 35 (26–55) | 49 (39–59) | < 0.001 |
| Sex (male) | 14 (45.2) | 29 (32.2) | 43 (35.5) | 45 (38.8) | 0.604 |
| Hypertension | 11 (35.5) | 22 (24.4) | 33 (27.3) | 46 (39.7) | 0.03 |
| Diabetes mellitus | 12 (38.7) | 9 (10.0) | 21 (17.4) | 43 (37.1) | < 0.001 |
| Cholesterol | 6 (19.4) | 7 (7.8) | 13 (10.7) | 10 (8.6) | 0.627 |
| Obesity | 13 (41.9) | 40 (44.4) | 53 (43.8) | 32 (27.6) | 0.014 |
| BMI (kg/m^2^) | 24.2 (22.0–29.4) | 24.7 (22.2–29.4) | 24.7 (22.2–29.4) | # |  |
| Cardiac disease | 6 (19.4) | 5 (5.6) | 11 (9.1) | 6 (5.2) | 0.265 |
| Vascular disease | 5 (16.1) | 0 (0.0) | 5 (4.1) | 0 (0) | 0.029 |
| Malignancy | 3 (9.7) | 2 (2.2) | 5 (4.1) | 1 (0.9) | 0.116 |
| HIV | 4 (12.9) | 16 (17.8) | 20 (16.5) | 24 (20.7) | 0.74 |
| CD4 cell count | $ | 179 (88–235) | 130 (76–231) | 279 (160–492) | 0.013 |
| HIV viral load | $ | 171 (33–634 104) | 4439 (42–797 098) | < 1000 |  |
| TB |  |  |  |  |  |
| current | 2 (6.5) | 5 (5.6) | 7 (5.8) | 4 (3.4) | 0.426 |
| previous | 0 (0) | 5 (5.6) | 5 (4.1) | 9 (7.8) | 0.21 |
| either | 2 (6.5) | 10 (11.1) | 12 (9.9) | 13 (11.2) |  |
| Other lung disease | 6 (19.4) | 8 (8.9) | 14 (11.6) | 6 (5.2) | 0.198 |
| CTD | 0 (0) | 1 (1.1) | 1 (0.8) | 1 (0.9) | 0.961 |
| CKD | 1 (3.2) | 6 (6.7) | 7 (5.8) | 8 (6.9) | 0.686 |
| Smoking history | 12 (38.7) | 22 (24.4) | 34 (28.1) | 11 (9.5) | < 0.001 |
| Alcohol | 6 (19.4) | 19 (21.1) | 25 (20.7) | 4 (3.4) | < 0.001 |
| Pregnant | 1 (3.2) | 24 (26.7) | 25 (20.7) | 5 (4.3) | < 0.001 |
| Previous COVID–19 |  |  |  |  |  |
| confirmed | 1 (3.2) | 3 (3.3) | 4 (3.3) | N/A |  |
| suspected | 1 (3.2) | 2 (2.2) | 3 (2.5) | N/A |  |
| Vaccination |  |  |  |  |  |
| complete | 8 (25.8) | 14 (15.6) | 22 (18.2) | N/A |  |
| partial | 4 (12.9) | 6 (6.7) | 10 (8.3) | N/A |  |
| either | 12 (38.7) | 20 (22.2) | 32 (26.4) | N/A |  |
| **Clinical characteristics** |  |  |  |  |  |
| COVID-19 primary diagnosis | 31 (100.0) | 0 (0.0) | 31 (25.6) | 116 (100.0) |  |
| Symptom duration (days) | 3 (2–5) | 0 (0–2) | 1 (0–3) | 7 (4–7) | < 0.001 |
| Cough | 21 (67.7) | 22 (24.4) | 43 (35.5) | 88 (75.9) | < 0.001 |
| Hemoptysis | 0 (0) | 3 (3.3) | 3 (2.5) | 3 (2.6) | 0.932 |
| SOB | 22 (71) | 12 (13.3) | 34 (28.1) | 78 (67.2) | < 0.001 |
| Fever | 13 (41.9) | 17 (18.9) | 30 (24.8) | 67 (57.8) | < 0.001 |
| Sore throat | 9 (29) | 9 (10.0) | 18 (14.9) | 12 (10.3) | 0.33 |
| Loss of smell | 4 (12.9) | 0 (0.0) | 4 (3.3) | 11 (9.5) | 0.045 |
| Loss of taste | 5 (16.1) | 0 (0.0) | 5 (4.1) | 8 (6.9) | 0.325 |
| Diarrhoea | 2 (6.5) | 5 (5.6) | 7 (5.8) | 15 (12.9) | 0.05 |
| Abdominal pain | 2 (6.5) | 11 (12.2) | 13 (10.7) | 8 (6.9) | 0.327 |
| Chest pain | 4 (12.9) | 5 (5.6) | 9 (7.4) | 22 (19) | 0.007 |
| Headache | 3 (9.7) | 10 (11.1) | 13 (10.7) | 15 (12.9) | 0.551 |
| Malaise | 15 (48.4) | 15 (6.7) | 30 (24.8) | 16 (13.8) | 0.041 |
| Myalgia | 10 (32.3) | 5 (5.6) | 15 (12.4) | 29 (25) | 0.009 |
| **Laboratory values** |  |  |  |  |  |
| Oxygen saturation (%) | 94 (88–98) | 97 (96–99) | 97 (93–99) | 94 (90–97) | < 0.001 |
| PaO_2_ (kPa) | 9.3 (6.5–14.8) | 12.8 (10.2–16.8) | 11.7 (8.9–16.2) | 8.7 (7.2–11.9) | < 0.001 |
| FiO_2_ (%) | 40 (21–80) | 21 (21–21) | 21 (21–33) | 21 (21–40) | < 0.001 |
| P:F ratio | 170 (111–332) | 415 (287–539) | 360 (180–511) | 246 (137–357) | 0.004 |
| pH | 7.46 (7.40–7.49) | 7.41 (7.35–7.46) | 7.43 (7.37–7.47) | 7.45 (7.42–7.48) | 0.116 |
| Sodium (mmol/L) | 135 (132–137) | 136 (134–138) | 136 (133–138) | 136 (133–140) | 0.389 |
| Potassium (mmol/L) | 4.2 (3.9–4.9) | 4.3 (3.8–4.8) | 4.3 (3.8–4.8) | 4.2 (3.8–4.6) | 0.391 |
| Urea (mmol/L) | 6.3 (4.1–8.9) | 4.1 (2.8–7.0) | 4.8 (2.8–7.9) | 4.7 (3.4–9.2) | 0.514 |
| Creatinine (mmol/L) | 77 (56–91) | 68 (53–93) | 72 (54–92) | 75.5 (57.5–113.5) | 0.241 |
| WCC (× 10^9^/L) | 8.3 (6.1–11.7) | 8.9 (6.7–12.9) | 8.8 (6.3–12.7) | 7.6 (6.2–9.9) | 0.017 |
| Neutrophil count (× 10^9^/L) | 7.9 (4.6–9.9) | 6.7 (4.8–10.3) | 6.8 (4.7–10.0) | 6.0 (4.2–7.8) | 0.024 |
| Lymphocyte count (× 10^9^/L) | 0.9 (0.6–1.4) | 1.1 (0.7–1.5) | 1.1 (0.7–1.4) | 1.1 (0.8–1.7) | 0.202 |
| N:L ratio | 8.1 (4.8–15.0) | 7.4 (3.7–10.5) | 7.6 (4.2–12.0) | 5.5 (3.2–7.7) | 0.003 |
| Hemoglobin (g/dL) | 12.8 (10.2–14.0) | 11.2 (9.8–13.2) | 11.8 (9.8–13.6) | 12.5 (11.2–13.6) | 0.08 |
| MCV | 91.7 (84.8–95.5) | 91.8 (86.0–95.1) | 91.8 (85.8–95.3) | 88.7 (84.9–92.3) | 0.002 |
| Platelet count (× 10^9^/L) | 243 (177–468) | 230 (174–315) | 232 (174–330) | 257 (211–333) | 0.043 |
| CRP (mg/L) | 134 (45–221) | 66 (15–169) | 78 (20–190) | 138 (63–222) | 0.008 |
| LDH | 409 (313–627) | 454 (316–564) | 453 (307–582) | 446 (288–606) | 0.797 |
| ALT (U/L) | 27 (16–38) | 24 (12–38) | 25 (13–38) | 28 (17–46) | 0.219 |
| HbA1c (%) | 6.5 (6.0–8.0) | 5.6 (5.3–6.0) | 5.7 (5.4–6.5) | 7.8 (6.3–11.7) | < 0.001 |
| Total cholesterol | 4.4 (3.2–5.0) | 3.8 (3.5–4.9) | 4.1 (3.4–4.9) | 4.0 (3.0–4.9) | 0.975 |
| D-dimer (mg/L) | 1.5 (0.5–5.4) | 1.9 (1.0–3.0) | 1.8 (0.6–5.0) | 0.6 (0.3–1.1) | 0.029 |
| **Outcomes** |  |  |  |  |  |
| Highest level of care |  |  |  |  |  |
| Outpatient | 0 (0) | 0 (0) | 0 (0) | 0 (0) |  |
| General wards | 26 (83.9) | 77 (85.6) | 103 (85.1) | 74 (63.8) | < 0.001 |
| ICU/HCU | 5 (16.1) | 13 (14.4) | 18 (14.9) | 42 (36.2) | < 0.001 |
| Hospital stay (days) | 5 (3–7) | 7 (3–12) | 6 (3–10) | 6 (3–10) | 0.548 |
| Outcome |  |  |  |  |  |
| Survived | 27 (87.1) | 76 (84.4) | 103 (85.1) | 85 (73.3) | 0.024 |
| Deceased | 3 (9.7) | 9 (10.0) | 12 (9.9) | 31 (26.7) | 0.001 |
| Censored | 1 (3.2) | 5 (5.6) | 6 (5) | 0 (0) | 0.015 |
| Table values are number (percent) or median (interquartile range).  *Comparing fourth-wave cohort (all) and first-wave cohort  # No data  $ ≤ three values | | | | | |
| ALT = alanine transaminase, CKD = chronic kidney disease, CTD = connective tissue disease, CRP = C-reactive protein, FiO_2_ = fraction of inspired oxygen, HCU = high-care unit, HIV = human immunodeficiency virus, HbA1c = glycated hemoglobin, ICU = intensive-care unit, LDH = lactate dehydrogenase, MCV = mean corpuscular volume, N:L ratio = neutrophil-to-lymphocyte count ratio, P:F ratio = ratio of FiO_2_ to PaO_2_, PaO_2_ = partial pressure of oxygen, SOB = short of breath, TB = tuberculosis, WCC = white cell count | | | | | |
